# Anti-NMDA and Anti-AMPA Receptor Antibodies in Central Disorders: Preclinical Approaches to Assess Their Pathological Role and Translatability to Clinic

**DOI:** 10.3390/ijms241914905

**Published:** 2023-10-05

**Authors:** Guendalina Olivero, Alessandra Roggeri, Anna Pittaluga

**Affiliations:** 1 Department of Pharmacy (DiFar), University of Genoa, Viale Cembrano 4, 16148 Genoa, Italy; guendalina.olivero@unige.it (G.O.); alessandra.roggeri@unige.it (A.R.); 2 Center of Excellence for Biomedical Research, 3Rs Center, Department of Pharmacy (DiFar), University of Genoa, Viale Cembrano 4, 16148 Genoa, Italy; 3 IRCCS Ospedale Policlinico San Martino, Largo Rosanna Benzi 10, 16145 Genoa, Italy

**Keywords:** NMDA receptor, AMPA receptor, autoantibodies, synaptic transmission

## Abstract

Autoantibodies against NMDA and AMPA receptors have been identified in the central nervous system of patients suffering from brain disorders characterized by neurological and psychiatric symptoms. It has been demonstrated that these autoantibodies can affect the functions and/or the expression of the targeted receptors, altering synaptic communication. The importance to clarify, in preclinical models, the molecular mechanisms involved in the autoantibody-mediated effects has emerged in order to understand their pathogenic role in central disorders, but also to propose new therapeutic approaches for preventing the deleterious central consequences. In this review, we describe some of the available preclinical literature concerning the impact of antibodies recognizing NMDA and AMPA receptors in neurons. This review discusses the cellular events that would support the detrimental roles of the autoantibodies, also illustrating some contrasting findings that in our opinion deserve attention and further investigations before translating the preclinical observations to clinic.

## 1. Introduction

Defects in synaptic transmission are at the basis of many neurodegenerative and neuropsychiatric disorders, which are defined as “synaptopathies” [1]. These defects originate from either neuronal or non-neuronal events. Neuronal events are preferentially sustained by excessive glutamate signal or reduced GABAergic inhibitory synaptic plasticity [2] while the non-neuronal events originate from astrocytes, microglia, lymphocytes, and macrophages [3,4], and are mediated by cytokines, chemokines, complement, and autoantibodies.

In recent years, some central neurological disorders have been associated with the production of autoantibodies recognizing neuronal cell-surface antigens, such as receptors and ion channels, most of which are involved in synaptic transmission [5,6]. Although the mechanisms leading to the central pathological accumulation of autoantibodies remain unknown, tumors, viral infections, or genetic attitude are proposed to play a role. The immune responses leading to their overproduction probably start in the periphery, and then progress centrally, as suggested by the high levels of autoantibodies and antibody-producing cells in the central nervous system (CNS) at the acute stage of the disease. Furthermore, although autoantibodies could be either protective or pathogenic [7], the responsiveness of patients to immunotherapy and/or plasmapheresis is better interpreted by assuming that they preferentially play a pathogenic role in disease progression [8]. Antibodies’ overproduction typifies central immunocompetent diseases including “autoimmune encephalitis” and “autoimmune dementia”, but it is also detected in neurodegenerative disorders including Alzheimer’s disease (AD), Lewy body dementia (LBD), frontotemporal dementia (FTD), and prion diseases.

Based on the knowledge so far available, it is important, from a clinical point of view, to obtain an early diagnosis of the overproduction of autoantibodies, to assure a rapid intervention but, concomitantly, it is also crucial to clarify the molecular/cellular events involved in the antibody-mediated synaptic deterioration. Defining these events would improve our knowledge of the etiopathogenesis of certain central disorders, but also might suggest new therapeutic approaches for their clinical management.

Hereafter, we will review some of the available data from preclinical studies concerning the impact of antibodies recognizing NMDA and AMPA receptors in neurons. This review is dedicated to comparing the results concerning the impact of autoantibodies and of commercial antibodies on NMDA and AMPA receptor distribution in synaptic membranes and on their efficiency in controlling synaptic communication, with particular attention to the glutamatergic transmission. The revised results support the pathological roles of the autoantibodies, but also illustrate some contradictory findings that in our opinion deserve further attention before translating the preclinical observations to clinic.

## 2. NMDA Receptors and Anti-GluN Autoantibodies in Central Disorders

NMDA receptors are ionotropic glutamate receptors composed of the tetrameric assembly of the obligatory GluN1 subunits, which associate with the GluN2 (A-D) or the GluN3 (A-B) subunits or both [9]. The stoichiometry of the subunit assembly defines the NMDA receptors’ properties, including the affinity of the receptor for the two main natural agonists (i.e., glutamate and glycine), as well as the permeability of the associated channel to Mg^2+^ ions, also determining the channel gating [10]. NMDA receptors have a wide distribution in the CNS since they are located in neurons, astrocytes, and oligodendrocytes as well. Several reviews have been dedicated to summarizing the main features and the role of NMDA receptors in physiological conditions and in neurological disorders and these aspects will not be discussed further here (but please refer to [10,11,12,13]).

Autoantibodies against NMDA receptors were first identified in the cerebrospinal fluid (CSF) and in the sera of patients suffering from anti-NMDA receptor encephalitis, one of the most common forms of autoimmune encephalitis, and proposed as specific markers of this disorder [14]. The conclusion is supported by the correlation linking the endogenous production of the autoantibodies (i.e., specifically of the anti-GluN1 autoantibody) and the progression of the disease [15,16,17,18,19]. Anti-NMDA receptor encephalitis is typified by neuropsychiatric symptoms similar to those caused by non-competitive NMDA antagonists (i.e., ketamine or phencyclidine), including psychosis, cognitive impairment, anxiety, irritability, and autonomic disorders, but also seizures, catatonia, and coma. Conventional MRI images usually do not show any abnormalities in the brain and in about 80% of patients the disease recovers after immunotherapy and/or plasmapheresis, well in line with the pathogenetic role of the autoantibodies. In the case of delayed treatments, the symptoms even worsen, suggesting the need for rapid therapies of intervention [20,21,22]. First associated with the presence of ovarian teratoma, it is nowadays recognized that anti-NMDA receptor encephalitis could also be secondary to viral infections or could have other, so far unknown, etiology. In most cases, the triggering event starts in the periphery, and then progresses centrally, where it is sustained by a robust intrathecal synthesis of anti-GluN1 antibodies [14].

Autoantibodies targeting NMDA receptors were also detected in patients suffering from AD and Parkinson’s disease (PD), from dementia and epilepsy, and in schizophrenic individuals [23,24].

The identification of NMDA receptor autoantibodies in the serum of psychotic patients supports the hypothesis of an autoimmune origin for certain idiopathic forms of this central disorder [25,26,27]. The hypothesis, however, needs confirmation since the endogenous level of the autoantibodies is highly variable among individuals, and in most cases, of modest entity. Furthermore, the nature of the Ig involved is a matter for discussion. In 10% of schizophrenic patients, the antibodies which target the GluN1A, but also the GluN2B subunit, were mainly identified as IgA and IgM, and, to a lesser extent, as IgG [28], but only the IgG anti-NMDA receptor antibodies were reported to decrease the density of the synaptic and extra-synaptic NMDA receptor and/or to alter their neuronal functions [29].

Autoantibodies targeting NMDA receptors are also associated with an atypical form of autoimmune dementia, characterized by psychiatric features, which are absent in the classic neurodegenerative dementia, and that benefits from immunotherapy. NMDA receptor antibodies consisting of IgM, IgA, or IgG subtypes were detected in a significant percentage of patients with dementia but not in cognitively healthy controls, but again, their role in clinical manifestation remains, so far, elusive [30,31].

The prevalence of anti-NMDA receptor autoantibodies in AD and PD patients is low when compared to that detected in subjects suffering from atypical dementia, suggesting that in the former diseases, the autoantibodies would not be pathogenetic, per se, but rather modulate the phenotype of the pathologies [32]. To note, although their overproduction in AD patients is associated with an increased rate of psychotic symptoms [32], it is proposed that anti-NMDA autoantibodies could mediate protective innate responses by increasing the resilience to central injures and reducing the excitotoxicity elicited by an excessive NMDA receptor activation [33]. The hypothesis is attractive and well consistent with the finding that low levels of anti-NMDA receptor autoantibodies are also detectable in healthy people [34].

## 3. AMPA Receptors and Anti-GluA Autoantibodies in Central Disorders

AMPA receptors mediate the fast excitatory transmission and are pivotal to synaptic plasticity. They are widely distributed in the CNS and are composed of four subunits, namely GluA1, GluA2, GluA3, and GluA4, encoded by different genes, with a 70% sequence homology, that assemble in tetrameric complexes with either homomeric or heterodimeric composition [35,36,37].

AMPA receptors traffic in and out of synaptic and/or extra-synaptic membranes in resting conditions (with a half-life of a few minutes), as well as in response to neuronal activity [38,39,40,41], but also move laterally between the synaptic and the extra-synaptic membrane compartments [40,42]. All these events control the synaptic plasticity and dictate the physio-pathological role of these receptors in the mammal’s brain.

Several CNS disorders have been associated with the overproduction of autoantibodies recognizing GluA subunits. The first disease was Rasmussen’s encephalitis [43] which is a severe and chronic brain disorder mainly affecting children or young adolescents. It is characterized by frequent focal seizures that are refractory to antiepileptic drugs, and that can progress to “epilepsia partialis continua” [44,45]. Untreated patients develop hemiparesis, hemianopia, and neurological and cognitive decline. The only cure is surgery [46,47,48] that, however, causes severe neurological deficits. In a few cases, plasmapheresis reduces status epilepticus [49,50], supporting an immune-mediated pathogenesis of this encephalitis. Accordingly, in 1994, Rogers and colleagues observed recurrent seizures and cortical inflammation in rabbits with a high titer of circulating anti-GluA3 antibodies and, indeed, anti-GluA3 antibodies were detected in the sera of patients suffering from this encephalitis, whose clinical gravity was reduced via plasmapheresis [50].

Anti-GluA3 autoantibodies are also detected in biological fluids from patients suffering from FTD. This is the third-most common form of dementia in the elderly [51], and it is characterized by the atrophy of the frontal and temporal lobes, subcortical gliosis, and neuronal loss. The pathology is typified by the dysregulation of serotonin, dopamine, GABA, and above all, glutamate innervation [52], as well as changes in AMPA and NMDA receptors [53] and altered AMPA receptor subunit composition at the synaptic level [54]. The course of the pathology is progressive, results in cognitive impairment and motor deficits, and often has a lethal outcome. Starting from the early 2000s, the FTD pathogenesis was proposed to involve autoimmunity [55,56,57]. Specifically, Borroni and colleagues identified the presence of anti-GluA3 antibodies in the serum and in the CSF of a significant number of patients suffering from FTD [58]. The authors also reported a deep alteration of the AMPA receptor-mediated neuronal functions, well consistent with the cognitive impairment experienced by the FTD patients that further strengthens the hypothesis of an autoimmune nature of the disease.

The anomalous production of anti-AMPA receptor autoantibodies in humans correlates not only with ongoing neuronal disorders but also represents a side effect of therapies. It is the case of multiple sclerosis and of the approved disease-modifying drug alemtuzumab, a humanized monoclonal antibody introduced in 1991 for the treatment of patients with secondary progressive MS and now approved for the treatment of the relapsing remitting form of multiple sclerosis as a second-line therapy. The monoclonal antibody binds the CD52 proteins on lymphocytes, monocytes, macrophages, eosinophils, and dendritic cells, targeting these cells for destruction, resulting in a selective depletion of circulating B-cells and T-cells [59]. Some of the adverse effects elicited by the monoclonal antibody therapy, particularly two cases of secondary autoimmune encephalopathy observed in MS patients after the alemtuzumab infusion [60,61,62], correlate with clinical signs observed in patients [60] with a high titer of circulating anti-AMPA receptor autoantibodies. In both cases, the autoimmune encephalitis emerged as a polymorphic epilepsia partialis continua, status epilepticus and progressive aphasia, were typified by an MRI pattern compatible with encephalitis and, as a matter of fact, the analysis of the CSF and the serum unveiled a high titer of antibodies against the GluA3 subunit. The immunoglobulin treatment ameliorated the neurological condition, consistent with the conclusion that alemtuzumab could have primed an autoimmune encephalitis involving antibodies directly against the AMPA receptors.

## 4. Pathogenic Mechanisms of Neuronal Cell-Surface Autoantibodies

Before discussing the molecular events proposed to account for the pathogenetic effects of anti-receptor autoantibodies, it is important to remind that the presence of autoantibodies in the CSF/serum does not “per se” imply a pathological condition. Indeed, autoantibodies are also found in the CSF of healthy people, usually at very low concentrations, and could have a physiological role [63]. Rather, the involvement of autoantibodies in disease progression is positively associated with their increased production, which correlates to the gravity of the clinical symptoms (as the gravity becomes worse, the antibody titer increases).

The first aspect to be verified when investigating the impact of an autoantibody in the course of a disease is its pathogenic activity. This can be achieved by using in vitro models that would permit the verification of the efficiency of the autoantibody to modify the functions of selected membrane protein(s), and to trigger detrimental cellular responses that could subserve and sustain some of the in vivo clinical symptoms in patients.

The data so far available highlighted the following principal mechanisms through which antibodies can affect the receptor-mediated responses [64,65].

Autoantibodies can:

(i) Modify the in–out receptors’ movements in plasma membranes by either stabilizing the receptor protein in the phospholipid bilayer or, alternatively, by increasing its endocytosis and subsequent association with cytosolic proteins (i.e., beta-arrestin), which would favor protein degradation [18]. In the first case, antibodies would stabilize the receptors in membranes and potentially amplify the associated signal. Differently, in the second case, they would reduce the number of receptors and accelerate their endocytosis and cytosolic degradation, causing a loss of function and a concomitant receptor depletion in the targeted cells. To note, the mechanisms of the in–out trafficking of proteins in neuronal plasma membranes control the density and therefore the signals of several receptors, including NMDA receptors [66], AMPA receptors [67] but also GABA_B_ receptors [68] and glycinergic receptors [69]. Therefore, by interfering with the trafficking of these receptors, autoantibodies would alter the efficiency of either the excitatory or the inhibitory synaptic plasticity, exposing the brain to a detrimental condition;
(ii) Behave either as agonists or antagonists, or even imitate allosteric ligands, influencing the responsiveness of the receptors toward the endogenous ligand(s) [70,71]. As a matter of fact, although the available literature is more consistent with an antagonistic profile [72], data also exist supporting an agonist-like activity in neurons [73];
(iii) Disrupt the protein–protein interactions that control the strength of the synaptic connections, affecting the stability of the synapses themselves. This is the case of LGI1 antibodies which block the interaction with LGI1 with the ADAM22/23 proteins, destabilizing the synaptic active zone and leading to the decreased surface accumulation of the AMPA receptors [74].

In addition to these mechanisms, the presence of autoantibody–antigen immunocomplexes in plasma membranes can also activate the complement cascade through the C1q-mediated classical pathway. The classical pathway is initiated by complement component C1q which is composed of six identical subunits with globular heads and acts as an initial pathogenic sensor. C1q binds the antibody–antigen complexes and activates an enzymatic pathway that cleaves complement proteins to produce opsonins that either promote the lysis of the cells bearing the antibody–antigen complex [75] or even modify some cellular functions, including the efficiency of the glutamate release [76]. For instance, the presence of the anti-CCR5-CCR5 receptor complex in the plasma membranes of cortical glutamatergic nerve endings was reported to trigger the activation of the complement through the classical C1q-mediated antibody-dependent pathway, leading to an abnormal release of glutamate, deleterious to CNS [77].

Once the in vitro studies highlight some of the cellular and molecular events triggered by the autoantibodies that could be pathogenic, these observations must be translated to in vivo animal models (i.e., via the passive transfer of the autoantibodies purified from the sera of patients, or by inducing an active immunization in animals) in an attempt to verify whether the presence of circulating antibodies can reproduce some of the disease symptoms and, in the positive, if the gravity of these symptoms correlates to the amount of the circulating antibody [78].

## 5. Cellular and Molecular Mechanisms Mediating the Impact of Anti-NMDA Receptor Antibodies in Neurons: Preclinical Results in Animals and Transability to Human

### 5.1. Impact of Anti-NMDA Receptor Autoantibodies in Neurons

In the last twenty years, several studies have been dedicated to clarifying the molecular mechanisms involved in the anti-GluN autoantibody-mediated modulation of NMDA receptors and synaptic transmission [79,80,81]. To this aim, the CSF, sera, or specific human anti-NMDA autoantibodies isolated from patients suffering from encephalitis or psychosis [15,16,81,82], as well as human-derived anti-GluN1 monoclonal antibodies produced by human memory B-cells [19], were analyzed in in vitro models (brain slices, cultured hippocampal neurons, cultured HEK cells) to highlight neuronal changes that could determine the symptoms that typify autoimmune neuropathies in humans [5].

The attention mainly focused on the GluN1 subunit. Despite the widespread distribution of NMDA receptors in the CNS, it emerged that human anti-GluN1 IgG autoantibodies isolated from patients’ CSF preferentially bind the NMDA receptors in the hippocampal regions [17,79,80]. Human anti-GluN1 autoantibody immunostaining was superimposable to that elicited by a commercial anti-GluN1 antibody, used in this case to confirm the labeling of the NMDA receptor complex [15,17,79]. Similarly, in cultured HEK cells, the human anti-NMDA receptor autoantibody immunopositivity was superimposable to that obtained with a mixture of commercial anti-GluN1 and anti-GluN2 antibodies [16]. To note, while the commercial antibodies were raised against the COOH tail of the receptor subunit, the autoantibodies recognized the NH_2_ terminal domain of the GluN protein.

In general, patients’ CSF or specific human anti-NMDA receptor autoantibodies caused a significant and diffuse internalization of the NMDA receptor complex that emerged as a significant reduction in both the GluN1 and the GluN2 subunits in the plasma membranes [15,17]. Internalization was proposed to depend on an antibody-induced desynchronization of the events that assure the insertion of the GluN subunits (and therefore of the NMDA receptors) in membranes. Specifically, the anti-NMDA receptor autoantibodies were shown to interfere with the ephrin-B2 receptor, a tyrosine kinase fundamental for the stabilization of NMDA receptors in membranes [82,83] at both synaptic and extra-synaptic sites. The hypothesis was confirmed by in vivo results showing that the passive transfer in rodents of human anti-NMDA receptor encephalitis patients’ CSF or of human anti-NMDA receptor autoantibodies elicited depression-like behaviors and memory deficits, which recovered following the administration of ephrin-B2 [84,85,86] or of selective NMDA receptor positive allosteric modulators [87].

The autoantibodies isolated from the sera of patients suffering from anti-NMDA receptor encephalitis did not affect the number of synaptic contacts, dendritic branching, and spines [15]. Rather, the autoantibodies specifically decreased the NMDA-mediated synaptic currents and reduced long-term potentiation in rat and mouse hippocampal slices, suggesting a preferential postsynaptic site of action of the antibody [15,16]. Notably, these observations were replicated in rats administered with the CSF from patients with a high titer of anti-GluN1 autoantibodies. In these animals, the reduced clustering of postsynaptic NMDA receptors in the hippocampus was comparable to that detected in the autoptic samples from patients with anti-NMDA receptor encephalitis [15].

Recently, neuronal exosomes enriched with NMDA receptor subunits have been found in the serum and CSF of patients suffering from encephalitis. Sera collected from mice immunized with these exosomes showed increased levels of antibodies recognizing NMDA receptors when compared to the control animals, suggesting a possible role of the extracellular vesicles in stimulating the immune response [88].

In general, the available data in the literature agree with the conclusion that the amino terminal domain (ATD) of the GluN1 subunit represents the preferential binding site of human anti-NMDA receptor autoantibodies. The GluN1 ATD exists in different splice variants that dictate the presence of the N1 cassette [36]. The N1 cassette controls the sensitivity of the receptor to protons, spermine, zinc ions, and glutamate as well as the time of deactivation of the NMDA receptor complex [9]. Anti-GluN1 antibodies from patients suffering from NMDA receptor encephalitis were reported to bind the ATD of the GluN1 subunit independently from the presence or the absence of the N1 cassette. Rather, the preferential target of these autoantibodies was proposed to correspond to a small epitope of the amino-terminal domain of the GluN1 receptor subunit that includes the amino acid 368, since anti-NMDA receptor autoantibodies do not bind to a 368 mutated GluN1 subunit protein [89].

### 5.2. Impact of Commercial Anti-NMDA Receptor Antibodies in Neurons

The data obtained with human autoantibodies were replicated by using commercial IgG antibodies raised against the ATD of the GluN1 but also of the GluN2 subunits. As already introduced, the immunostaining elicited by commercial antibodies (i.e., recognizing the internal COOH terminus) was superimposable to that detected with anti-GluN1 autoantibodies [15]. Furthermore, anti-GluN1 commercial antibodies recognizing the NH_2_ terminus elicit functional adaptations largely comparable to those induced by human anti-GluN1 autoantibodies [70].

Experiments were carried out in glutamatergic nerve endings isolated from the hippocampus of healthy adult mice (i.e., the synaptosomes, [90]). These particles are endowed with NMDA autoreceptors that consist of GluN1, GluN2A, and B, and also include GluN3A subunits. The activation of these receptors favors the presynaptic release of glutamate [10,40,91,92]. The acute incubation (30 min) of hippocampal glutamatergic synaptosomes with commercial anti-GluN1, anti-GluN2Aandr B, or anti-GluN3A antibodies drastically reduced the NMDA-mediated facilitation of the glutamate release. To note, the specificity of the antibody-mediated effects was proven by the results obtained with a selective anti-GluN3B antibody. The GluN3B subunit does not participate in the expression of the hippocampal NMDA autoreceptors, and, therefore, the anti-GluN3B antibody would not be expected to affect the NMDA-evoked releasing activity, as indeed it was observed [70]. The loss of function elicited by the commercial antibodies relied on a significant internalization of the NMDA receptor complex, which emerged in biotinylation studies as a reduced insertion of both the GluN1 and the GluN2B subunits in synaptosomal membranes [70]), instead of a receptor deactivation, as first proposed by Zhang and colleagues [16]. To note, the exposure to anti-GluN1 or anti-GluN2A/B antibodies did not modify the viability of the cortical synaptosomes, as already reported by Hughes and colleagues in cultured neurons [15]. Furthermore, the presence of the antibody/antigen complex did not induce complement-mediated cytotoxic responses, well in line with the proposed internalization of the anti-GluN antibody/GluN subunit complex, that would prevent the activation of the complement-mediated classical pathway and the consequent opsonization of nerve terminals (please compare with [77]).

The large consistency in the biochemical and functional results obtained with the commercial anti-GluN antibodies allows, in our opinion, to propose the use of commercial anti-GluN antibodies as a useful experimental tool to analyze the interaction of the autoantibodies with NMDA receptors and to investigate the functional consequences elicited by the presence of the antibody/antigen complex in neurons.

### 5.3. Impact of Anti-NMDA Receptor Autoantibodies in Neurons: Future Perspectives

An aspect so far poorly investigated is whether human anti-NMDA antibodies target indiscriminately all the NMDA receptors or, rather, if they specifically interact with selected NMDA receptor subtypes in selected subpopulations of nerve endings (i.e., the glutamatergic, the GABAergic, the dopaminergic, or the noradrenergic ones, see [10]). The question deserves attention since, in the positive, the specificity would provide a rationale for selected synaptic alterations that might determine the pathological phenotype of central disorders. For instance, anti-NMDA receptor autoantibodies that could specifically target and internalize NMDA heteroreceptors controlling GABA release would reduce the central GABAergic tuning, favoring the synaptic impairments thought to underlie the schizophrenic profile [93,94]. In this view, the human anti-NMDA receptor autoantibodies’ effects were proven not to be restricted to excitatory synapsis but also to occur in inhibitory neurons, as already discussed in the literature [17].

Another aspect that would deserve further attention is the possibility that anti-GluN autoantibodies can indirectly influence non-NMDA receptors colocalized and functionally associated with the NMDA receptors, altering the mechanism of metamodulation of synaptic transmission [39,95]. This possibility is particularly intriguing since defects or changes in receptor metamodulation are proposed to subserve neuronal vulnerability. It is the case with dopaminergic type 1 receptors (D1Rs), that colocalize with NMDA receptors and are functionally regulated by human anti-NMDA receptor antibodies which, by binding the NMDA receptor counterpart, alter the NMDA receptor/D1R receptor–receptor interaction [96]. In this view, the synaptosomes could provide a useful experimental tool to investigate whether and how anti-GluN antibodies (the human antibodies but also the commercial ones) interfere with the NMDA receptors-mediated metamodulation of non-NMDA receptors in specific subpopulations of nerve endings.

The results described in this chapter are summarized in Table 1.

## 6. Cellular and Synaptic Mechanisms Mediating the Impact of Anti-AMPA Receptor Antibodies in Neurons: Preclinical Results in Animals and Transability to Human

The available information concerning the effects elicited by human anti-AMPA receptor autoantibodies in the CNS was obtained via experimental approaches comparable to those applied to the anti-NMDA receptor antibody-mediated effects. Also, in this case, the CSF, the serum, or some purified human anti-GluA antibodies from patients suffering from central anti-AMPA autoimmune pathologies (i.e., Rasmussen encephalitis; epilepsy; FTD), as well as purified anti-GluA antibodies isolated from animals immunized with selected GluA aminoacidic sequences (i.e., GluA1, 2, and 3 subunits; [98,99]), were tested in in vitro and ex vivo neuronal models (i.e., HEK cells, cultured neurons, isolated nerve terminals, brain slices) to evaluate changes in the AMPA receptor distribution and functions [98,100,101,102,103,104,105]. In some cases, the study was translated to in vivo protocols to compare the changes in behavioral skills detected in animals with the clinical signs in the donor patients [106].

The results highlighted significant adaptations of the AMPA receptors elicited by anti-AMPA receptor antibodies that, however, were characterized by a marked variability. Heterogeneity might depend on the autoantibodies (i.e., antibodies purified from the CSF or the serum of patients suffering from Rasmussen’s encephalitis, FTD, or autoimmune encephalitis, as well as antibodies isolated from the CSF of animals immunized with a GluA antigen), on the CNS regions under study, on the experimental conditions applied (the titer and the time of exposure to the autoantibodies, the animal models), and on the targeted subunit (GluA3 or GluA1 and 2). Whatever the reason, the heterogeneity predicts a complexity of the molecular events associated with the antibody/antigen interaction that needs further investigation to be appropriately addressed.

### 6.1. Impact of Anti-AMPA Receptor Antibodies in Neurons: The Anti-GluA3 Antibodies

The research first focused on autoantibodies specific to the GluA3 receptor subunit isolated from patients suffering from Rasmussen’s encephalitis [49,73]. The results were consistent with an anti-GluA3 antibody-induced amplification of the AMPA receptor-mediated signal, an event that would account for the excitotoxic effects elicited by anti-AMPA receptor autoantibodies in neurons [105]. Specifically, in cultured mice neurons, an autoantibody raised against a subregion of the outer NH_2_ sequence of the GluA3 subunit (termed the GluR3B sequence, amino acid 372–395) caused a reversible, voltage-dependent activation of the AMPA receptors that was abolished by the selective AMPA antagonist CNQX. It was proposed that the antibody did not behave as a pure orthosteric agonist, but rather as an allosteric stabilizing agent, able to modify the configuration of the receptor and of the associated channel, favoring its activation [98,105].

To complicate the scenario, Day and colleagues recently demonstrated that a purified anti-GluA3B autoantibody increases, instead of inhibits, AMPA-mediated responses. The authors immunized rabbits with the GluR3B peptide and purified the protein A total IgG fraction which revealed a strong immunogenic response to the immunizing peptide [73,107,108]. The purified anti-AMPA receptor antibody bound specifically to the AMPA receptors in hippocampal neurons, with an efficiency comparable to that elicited by a commercial anti-AMPA receptor antibody. Differently from what was observed in cortical neurons, it reduced the mean synaptic excitatory postsynaptic currents’ frequency. The effect was long-lasting over time since it was observed after an acute short or long (30 min to 24 h) exposure to the antibody. The authors speculated a presynaptic locus of action for an anti-AMPA receptor antibody that could indirectly reduce the glutamate release or directly internalize the AMPA receptor.

In this context, we verify the impact of a commercial anti-GluA3 antibody recognizing the NH_2_ terminus of the receptor subunit on presynaptic release-regulating AMPA receptors. To this aim, we used mouse cortical synaptosomes, which are endowed with presynaptic release-regulating GluA3/GluA2 AMPA receptors that constitutively traffic in and out of the synaptosomal plasma membranes [40,104,109], and therefore provide an appropriate model for studying the impact of anti-GluA antibodies on native neuronal AMPA receptors. Glutamatergic cortical synaptosomes were acutely exposed (30 min) to a commercial antibody recognizing the NH_2_ terminus of the GluA3 subunit [104]. The commercial anti-GluA3 antibody did not modify the spontaneous release of glutamate, but significantly amplified the AMPA receptor-evoked release of glutamate in a CNQX-sensitive manner, suggesting that it does not behave as an orthosteric agonist but rather as a positive allosteric ligand [73,105,107]. Specifically, the anti-GluA3 antibody caused a significant enrichment in the synaptosomal membranes of both the GluA2 and the GluA3 subunits (the insertion of the latter one prevailed over that of the former one). The finding is consistent with the results by Malina and colleagues in 2006 and suggests that the anti-GluR3B antibody-induced activation of AMPA receptors is not restricted to GluA3 homomeric receptors but also pertains to GluA2-GluA3 heteromeric AMPA receptors [98]. Furthermore, it supports the conclusion that the anti-GluA3 antibody modifies the subunit composition of AMPA receptors by increasing the insertion of the Ca^2+^-permeable subunit (i.e., the GluA3 one), an event that would favor the detrimental consequences of the overactivation of AMPA receptors. Although mouse hippocampal glutamatergic synaptosomes also possess presynaptic GluA2-containing AMPA receptors, we did not investigate, so far, the impact of anti-GluA3 antibodies on these receptors and could not predict whether the hippocampal receptors respond differently to the commercial anti-GluA3 antibodies in terms of the efficiency of the glutamate release.

To verify the transability of the results obtained via the commercial antibodies to the human anti-GluA3 autoantibody, experiments were carried out to verify the impact of human sera with a high titer of circulating anti-GluA3 autoantibodies. Thanks to a collaboration with Barbara Borroni and Fabrizio Gardoni, we had the possibility to test the impact of sera from patients suffering from FTD (with a high titer of anti-GluA3 autoantibody) on the presynaptic AMPA autoreceptors controlling glutamate exocytosis from mouse cortical synaptosomes. The exposure of synaptosomes to the patient’s serum caused a significant decrease in the releasing efficiency of the AMPA autoreceptors [103]. The loss of function positively correlated to the titer of the antibody, since it was almost undetectable with the sera with a very low content of the anti-GluA3 antibody. Taking into consideration that the commercial autoantibodies and the sera from patients suffering from FTD were tested in the same experimental paradigm, the most conservative hypothesis to account for the inconsistency in the results is that the serum might contain components other than the GluA3 antibody that could influence the stability of the anti-GluA3/AMPA receptor interaction, shifting the outcome from facilitation to inhibition. Indeed, Peng et al., in 2015, showed that a significant internalization of AMPA receptors in cultured hippocampal neurons exposed to a commercial anti-GluA2 antibody could only be observed when a secondary antibody was concomitantly added to stabilize the antigen/antibody complex [102]. In line with the reduced AMPA receptor-mediated releasing activity triggered by the FTD patient’s serum, the exposure of rat primary hippocampal neurons to the CSF of FTD patients with a high titer of circulating anti-GluA3 autoantibodies decreased the synaptic localization of the AMPA receptors and caused a concomitant loss of dendritic spines [58]. Similarly, in neurons differentiated from human pluripotent stem cells, patients’ CSF caused a significant reduction in the GluA3 subunit [58].

### 6.2. Impact of Anti-AMPA Receptor Antibodies in Neurons: The Anti-GluA2 Antibodies

The heterogeneity of the results in the literature regarding the impact of anti-AMPA receptor autoantibodies in neurons, however, is not limited to anti-GluA3 antibodies, but also concerns the impact of other anti-AMPA receptor antibodies, specifically the anti-GluA1 and the anti-GluA2 receptor antibodies, which are overexpressed in the CSF and the serum of patients suffering from anti-AMPA receptor encephalitis [101,110].

For homology, with the anti-GluA3 antibody, the anti-GluA1 and anti-GluA2 antibodies were predicted to alter the synaptic localization and the number of AMPA receptors, promoting neuronal disfunctions.

Gahring and colleagues [111] investigated the effect of the serum of a patient suffering from progressive sporadic olivopontocerebellar atrophy who exhibits high serum titers of IgM autoantibodies recognizing the neuronal GluA2 subunit. Glutamate receptor currents were activated in cultured mouse neurons by the anti-GluA2 IgM and the effect was sensitive to CNQX and prevented by a synthetic peptide corresponding to the specific epitope region of GluA2 (AA 369–393) that would buffer the antibody, minimizing its interaction with the receptor subunit.

Differently, Balice-Gordon’s group (2015) [102] demonstrated that human anti-GluA1 and anti-GluA2 autoantibodies modify the distribution and the function of AMPA receptors. The autoantibodies increased the internalization of the synaptic AMPA receptors, then reduced the synaptic transmission, leaving an unaltered synapsis density and cell viability. The reduction in the excitatory transmission was paralleled by a concomitant compensatory decrease in the inhibitory transmission and by a later increased excitability, possibly to compensate for the loss of functions in the AMPA receptors. These results were largely reminiscent of those published by Lai and colleagues (2009) who demonstrated that anti-GluA1 and GluA2 antibodies significantly decreased the number of GluA2-containing AMPA receptor clusters at synapses in cultured neurons [101].

Peng and colleagues did not observe significant changes in AMPA receptors’ distribution and functions in cultured hippocampal neurons exposed to commercial antibodies raised against a synthetic peptide corresponding to the N-terminal portion of a rat GluA1 subunit (aa 271–285) or of the GluA2 subunit (aa 175–430) [102]. The lack of efficiency of these commercial antibodies, specifically of the anti-GluA2 antibody, is in a way surprising, since the antibody binds to an immunogenic sequence aa 175–430 in the NH_2_ terminus of the subunit protein, i.e., the sequence homologous to that of the GluA3B protein, that is selectively targeted by anti-GluA2 antibodies which behave as receptor agonists [111]. The complexity of the scenario is further increased by the finding that (i) the addition of a secondary antibody to cross-link the primary anti-GluA2 antibody with the respective antigenic sequence caused a marked reduction in the AMPA receptor density in neuronal membranes and (ii) a commercial anti-GluA2 antibody recognizing the outer sequence of the protein significantly favored the insertion and releasing activity of the AMPA autoreceptor [104]. As a matter of fact, the incubation of synaptosomes with the anti-GluA2 antibody increased the insertion of the GluA2 subunit in membranes, but almost triplicated that of the GluA3 protein. The altered distribution of the two receptor subunits was paralleled by an increased efficiency of the AMPA-evoked releasing activity, largely comparable to that elicited by the anti-GluA3 antibody [104].

### 6.3. Impact of Anti-AMPA Receptor Antibodies in Neurons: The Anti-GluA1 Antibodies

Lastly, the lack of efficacy of commercial anti-GluA1 antibodies in modifying the AMPA receptor-mediated control of glutamate transmission described by Peng and colleagues [102] was confirmed in cortical glutamatergic synaptosomes incubated with a commercial anti-GluA1 antibody, although, in this case, the lack of efficacy could also be ascribed to the scarce participation of the GluA1 subunit in the subunit assembly of the AMPA autoreceptor [104].

### 6.4. Impact of Anti-AMPA Receptor Antibodies in Neurons: Role of Complement

Attention was also paid to the possible involvement of complement in the neuronal events triggered by human anti-AMPA receptor autoantibodies. This immune component was proposed to participate in the excitotoxic effect elicited by anti-GluA3 antibodies in neurons, based on the efficiency of the complement regulatory protein to modify the resistance of these cells to anti-GluA3 antibodies [100,112]. In line with this view, we demonstrated that the complement-evoked release of glutamate from mice cortical-isolated synaptosomes [76,77] was significantly amplified in synaptosomes bearing the anti-GluA3 antibody/GluA3-AMPA receptor complexes in synaptic plasma membranes [104]. The increased release efficiency of complement in these terminals relied on the activation of the classical pathway [113], since the removal of the C1q component nulled the anti-GluA3 antibody-mediated amplification of the releasing activity.

### 6.5. Impact of Anti-AMPA Receptor Autoantibodies in Neurons: Future Perspectives

The preclinical in vitro results so far described unveil a high heterogeneity in the anti-GluA antibody-induced events, which might depend on the experimental approaches, on the main features of the antibodies under study, as well as on the concomitant presence of other immune components (i.e., secondary antibodies, proteins that stabilize the antibody–antigen complex, complement proteins), that can influence the antibody/antigen-mediated effects. The complexity of these observations indicates the need to deepen the knowledge of the impact of human anti-GluA antibodies (purified from the CSF or obtained following immunization or viral transcription of animals) on AMPA receptors, to verify their pharmacological interaction with native receptors and to decipher the functional consequences in terms of receptor trafficking, changes in the subunit composition, and/or responsiveness to AMPA ligands.

The results described in this chapter are summarized in Table 2.

## 7. Conclusions

The literature concerning the central impact of anti-AMPA and anti-NMDA antibodies in preclinical studies is dramatically heterogenous, as emerges from the main findings described in this review.

**Figure 1:**
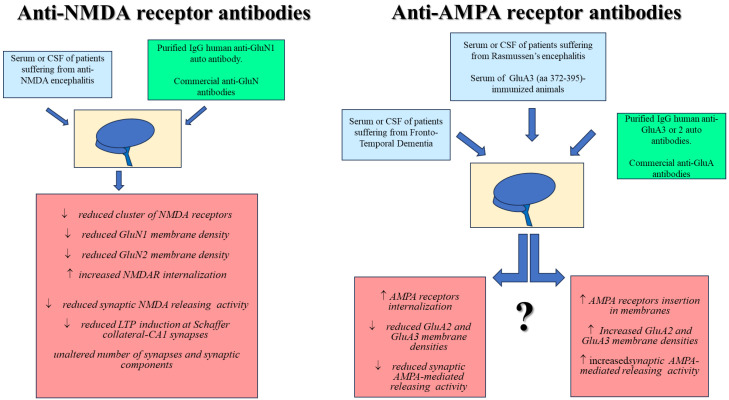
Role of anti-NMDA and anti-AMPA receptor antibodies on neuronal NMDA receptors (**left**) and AMPA receptors (**right**).

A key question concerns the impact of the antibodies on the receptor’s distribution and functions. In this regard, while there is a consensus on the impact of the anti-GluN antibodies on neuronal NMDA receptors, the role and the consequences elicited by the anti-GluA antibodies on neuronal AMPA receptors are far to be clarified and cannot be easily traced back to a common mechanism of action (Figure 1).

As far as the anti-GluN antibodies are concerned, the results are consistent with an antibody-induced internalization of the NMDA receptor subunit that well accounts for the synaptic desynchronization that supports some of the clinical manifestations observed in patients. The conclusion is confirmed by the significative overlapping of the effects exerted by anti-GluN autoantibodies that occur independently of the origin of the antibodies (i.e., antibodies isolated from the CSF, the serum of patients, human-derived anti-GluN1 monoclonal antibodies isolated from immunized animals, or produced from human CSF memory B-cells) and the different experimental models (cultured hippocampal neurons, HEK cells, mouse hippocampal synaptosomes). Interestingly, the effects elicited by the human anti-GluN antibodies are largely reproduced by commercial anti-GluN antibodies recognizing the NH_2_ terminus, opening the possibility to use these tools to decipher the cascade of events that lead to the internalization of the receptors, but also to investigate the responsiveness of the NMDA receptors that remain functionally available after the exposure to the antibody. The latter aspect has been, so far, poorly investigated but deserves, in our opinion, attention, since the synaptic derangements subserving the pathologic phenotype of patients suffering from the anti-NMDA receptor autoantibody-mediated disorders might be ascribed to the drastic reduction in the available NMDA receptors, but also to the so far unexplored structural and functional adaptation of the untargeted remaining NMDA receptors [25]. The results obtained in preclinical studies, in addition to being predictive of the cellular and molecular events that typify the course of autoimmune disorders, would also permit to check interventions for the management of these diseases (see, for instance, the selective positive allosteric modulator of NMDA receptors that reverses the memory and synaptic alterations caused by the CSF from patients with anti-NMDA receptor encephalitis in an animal model of the passive transfer of antibodies [87]). The possibility is attractive taking into consideration the so far unmet need for molecules/therapeutics to control the central NMDA receptor-related pathologies.

Differently, the results obtained with the anti-GluA autoantibodies are hugely heterogenous and more investigations are needed to clarify their role in dictating the changes in AMPA receptor expression and functions and, in general, in the cellular and molecular events supporting the anti-AMPA-induced central pathologies. The data available in the literature unveil opposite outcomes (i.e., facilitation or inhibition) that apparently are induced by similar antibodies (please compare [105] and [108]) in different tissue paradigms. The heterogeneity of the data suggests a need for the standardization of the techniques applied to immunize animals and to isolate and purify the autoantibodies.

## Figures and Tables

**Table 1:** Impact of anti-NMDA antibodies in the CNS: preclinical evidences.

Source	Targeted GluN Subunit	Experimental Paradigm	Effects	Reference
Serum and CSF of patients suffering from encephalitis Purified IgG human anti-GluN1 auto-antibody	GluN1	HEK293 cells transfected with rodent (or human) GluN1 or GluN2 (A, B, C, or D) subunits. Cultured rat hippocampal neurons	Immunolabeling of NMDA receptor clusters in postsynaptic dendrites ↓ Reduced cell-surface fraction of NMDA receptors	[80]
CSF from patients suffering from anti-NMDA receptor encephalitis Purified IgG human anti-GluN1 autoantibody	GluN1	Cultured rat hippocampal neurons	↓ Reduced cluster of NMDA receptors ↓ Reduced GluN1 density ↓ Reduced GluN2 density ↓ Reduced synaptic NMDA currents Unaltered number of synapses and synaptic components	[15]
CSF from patients suffering from anti-NMDA receptor encephalitis CSF from non-encephalitis patients (control CSF) Commercial anti-GluN1 and anti-GluN2B antibodies	GluN1	Transfected HEK293 cells expressing GluN1/GlUn2A or GluN1/GluN2B heteromers Cultured rat hippocampal neurons C57JBl mouse hippocampal slices	Anti-NMDA autoantibody-induced staining of NMDA receptors confirmed with commercial anti-GluN1 and anti-GluN2B Staining of native NMDARs in rat hippocampal neurons ↓ Reduced LTP induction at Schaffer collateral-CA1 synapses in mouse hippocampal slices.	[16]
CSF from patients suffering from anti-NMDAreceptor encephalitis Commercial anti-GluN1 and anti-GluN2B antibodies raised against the COOH terminus	GluN1	Transfected HEK293 cells expressing GluN1/GlUn2A or GluN1/GluN2B heteromers Whole rat brain lysate and rat cortical, hippocampal, and cerebellar lysates	Patients’ CSF immunoprecipitates GluN1 proteins from whole rat brain lysate and from cortical, hippocampal, and cerebellar lysates Patients’ CSF labels NMDA receptor in transfected cells In transfected HEK cells, the short exposure (2 min) to glutamate/glycine plus patient’s CSF prolongs the open duration of the NMDA receptor-associated channel. Differently, the prolonged exposure (>24 h) decreases NMDA receptors	[89]
CSF from patients suffering from anti-NMDA receptor encephalitis Purified IgG human anti-GluN1 antibody	GluN1 GluN2A GluN2B	Cultured rat hippocampal neurons	↓ GluN2-containing NMDA receptors density ↓ LTP induction at Schaffer collateral-CA1 synapses in mouse hippocampal slices ↑ Patient’s CSF increases the mobile fraction of GluN2A-NMDA receptors but decreases the GluN2B-NMDA receptors. Patient’s IgG disrupts GluN2A-NMDA receptor/EPHB2R interaction	[82]
CSF from patients suffering from anti-NMDA receptor encephalitis Commercial anti-GluN1 antibody	GluN1	Cultured rat hippocampal neurons	Patient and commercial anti-GluN1 antibody have comparable distribution and immunostainings ↓ Surface NMDA receptors in both excitatory and inhibitory hippocampal neurons ↑ NMDA receptor internalization ↓ NMDA receptor-mediated current amplitude	[17]
Recombinant monoclonal GluN1 antibody from cerebrospinal fluid memory B-cells	GluN1	Transfected HEK293 cells expressing GluN1/GluN2A or GluN1/GluN2B heteromers Cultured mouse hippocampal neurons Mouse brain slices	Staining of native GluN1-containing NMDA receptors in transfected HEK293 cells, in primary mouse hippocampal neurons and in mouse brain ↓ Synaptic NMDA receptor currents elicited by recombinant monoclonal GluN1 antibody	[19]
Commercial anti-GluN1, GluN2A/B, GluN3A antibodies	GluN1, GluN2B	Mouse hippocampal synaptosomes	↓ NMDA receptors cluster ↓ GluN1 density ↓ GluN2 density ↓ NMDA-mediated glutamate exocytosis	[70]
GluN1 antibodies derived from patients with anti-NMDAR encephalitis	GluN1		Staining of mature hippocampal primary neuron in culture Miniature spontaneous calcium transients (mSCaTs) mediated via NMDARs at synaptic spines are not altered in pathogenic GluN1 antibody-exposed conditions. Calcium does not accumulate in neuronal spines following brief exposure to pathogenic GluN1 antibodies	[97]

**Table 2:** Impact of anti-AMPA antibodies in the CNS: preclinical evidences.

Source	Targeted GluA Subunit	Experimental Paradigm	Effects	Reference
Serum of rabbits immunized with a specific amino acid sequence of the GluR3 protein (the Glu3B peptide, aa 372–395) Purified IgG anti-GluA3 autoantibodies Serum from patients with active Rasmussen’s	GluA3	Cultured fetal mouse cortical neurons	↑ Currents in neurons, prevented by CNQX The GluR3B peptide specifically blocks the antisera- and IgG-evoked currents The anti-GluR3B antibody mimics the serum from immunized rabbit and the purified IgG.	[73]
Murine antibody recognizing a specific sequence of the GluR3 protein (the Glu3B peptide, aa 372–395)	GluA3	Mouse coronal slices of somatosensory cortex Primary hippocampal cultures	Immunostaining of neurons ↑ CNQX-sensitive currents in neurons GluR3B peptide-induced neuronal death reduced by CNQX	[105]
Anti-GluA3 IgG isolated from GluA3 (aa 246–455)-immunized rabbits	GluA3	Rat mixed cortical neuronal and glial cultures Whole cell recording in cultured rat cortical neurons	↑ Release of LDH from mixed cultures in a GIKY52466 or CNQX-independent manner The plasma from immunized animals does not evoke currents in cultured neurons	[112]
Purified rabbit antibody recognizing a specific amino acid sequence of the GluR3 protein (the Glu3B peptide, aa 372–395) Monoclonal mouse anti-GluA3B	GluA3	Rat brain homogenates Xenopus laevi oocytes expressing GluA3 homomeric or GluA2/A3 heteromeric AMPARs	Purified rabbit anti-GluA3 antibody elicits whole cell currents that were sensitive to CNQX	[98]
Murine anti-autoantibody recognizing the amino acid sequence of the GluR3 protein (the Glu3B peptide, aa 372–395)	GluA3	DBA/2J mice immunized with the GluR3B peptide that express circulating anti-GluA3B autoantibodies	Immunized DBA/2J mice were more susceptible to pentetrazol-induced seizures	[106]
Serum and CSF containing anti-GluA3 autoantibodies from patients suffering from FTD	GluA3	Rat hippocampal neuronal primary cultures differentiated neurons from human-induced pluripotent stem cells (hIPSC)	↓ GluA3 subunit synaptic localization of AMPA receptor in hippocampal neuronal primary cultures ↓ Dendritic spines AMPA receptor in cultured hippocampal neurons ↓ GluA3 subunit fraction in the postsynaptic fraction of cultured hIPSC	[58]
Serum of patients suffering from FTD-Tau neuropathology with a high titer of anti-GluA3 autoantibody	GluA3	Mouse cortical synaptosomes	The serum does not evoke glutamate exocytosis ↓ AMPA-evoked glutamate release	[103]
Commercial anti-GluA3 antibody	GluA3	Mouse cortical synaptosomes	↑ GluA2 and GluA3 subunits insertion in synaptosomal plasma membranes The antibody does not evoke glutamate exocytosis ↑ AMPA-evoked glutamate release	[104]
IgG Anti-GluA3 antibody from rabbit immunized with the GluR3B peptide	GluA3	Mouse whole brain lysates Primary hippocampal neurons	Anti-GluA3 immunostaining in the whole brain lysate ↓ excitatory postsynaptic currents (EPSCs) in primary hippocampal neurons	[108]
Serum of a patient suffering from progressive sporadic olivopontocerebellar atrophy Purified IgM anti-GluA2 autoantibodies	GluA2	Cultured fetal mouse cortical neurons	↑ Currents in neurons in a CNQX-sensitive manner Currents were blocked by a synthetic peptide corresponding to the specific epitope region of GluR2 (aa 369–393)	[111]
Purified IgM anti-GluA1 and GluA2 autoantibodies from the CSF of patients suffering from anti-AMPA receptor encephalitis Commercial anti-GluA1 and GluA2 antibody	GluA2	Primary rat hippocampal neuron and astrocyte cocultures	↓ Surface AMPA receptor protein and synaptic localization Unmodified glutamatergic synapse density and cell viability ↑ Internalization of AMPA receptor clusters ↓ Miniature excitatory postsynaptic currents (mEPSCs) ↓ Miniature inhibitory postsynaptic currents (mIPSCs) ↑Intrinsic neuronal excitability after 48 h of treatment with patient CSF Commercial anti-GluA2 antibody does not cause receptor internalization	[102]
Anti-GluA2 autoantibodies purified from patients suffering from limbic encephalitis	GluA2	Human embryonic kidney 293 (HEK293) cells	↓ GluA2-containing AMPA receptor clusters number at synapses with a smaller decrease in overall AMPA receptor cluster density	[101]
Commercial anti-GluA2 antibody	GluA2	Mouse cortical synaptosomes	↑ Insertion of GluA2 and GluA3 subunits in synaptosomal plasma membranes The anti-GluA2 antibody does not evoke glutamate exocytosis ↑ AMPA-evoked glutamate release	[104]
Purified IgM anti-GluA1 autoantibodies from the CSF of anti-AMPA receptor encephalitis patients Commercial anti-GluA1 antibody	GluA1	Primary rat hippocampal neuron and astrocyte cocultures	↓ Localization of synaptic surface AMPA receptor protein The anti-GluA1 antibody does not modify the glutamatergic synapse density and the cell viability ↑ Internalization of AMPA receptor clusters ↓ Miniature excitatory postsynaptic currents (mEPSCs) ↓ Miniature inhibitory postsynaptic currents (mIPSCs) ↑ Intrinsic neuronal excitability after 48 h of treatment with patient CSF Commercial anti-GluA1 antibody does not cause receptor internalization	[102]
Commercial anti-GluA1 antibody	GluA1	Mouse cortical synaptosomes	The anti-GluA1 antibody does not evoke glutamate exocytosis The anti-GluA1 antibody does not modify the AMPA-evoked glutamate release	[104]

## Data Availability

Not applicable.
